# Carbon, Nitrogen and Phosphorus Accumulation and Partitioning, and C:N:P Stoichiometry in Late-Season Rice under Different Water and Nitrogen Managements

**DOI:** 10.1371/journal.pone.0101776

**Published:** 2014-07-03

**Authors:** Yushi Ye, Xinqiang Liang, Yingxu Chen, Liang Li, Yuanjing Ji, Chunyan Zhu

**Affiliations:** 1 Institute of Environmental Science and Technology, College of Environmental and Resource Sciences, Zhejiang University, Hangzhou, China; 2 Zhejiang Province Key Laboratory for Water Pollution Control and Environmental Safety, Hangzhou, China; University of Maryland, United States of America

## Abstract

Water and nitrogen availability plays an important role in the biogeochemical cycles of essential elements, such as carbon (C), nitrogen (N) and phosphorus (P), in agricultural ecosystems. In this study, we investigated the seasonal changes of C, N and P concentrations, accumulation, partitioning, and C:N:P stoichiometric ratios in different plant tissues (root, stem-leaf, and panicle) of late-season rice under two irrigation regimes (continuous flooding, CF; alternate wetting and drying, AWD) and four N managements (control, N0; conventional urea at 240 kg N ha^−1^, UREA; controlled-release bulk blending fertilizer at 240 kg N ha^−1^, BBF; polymer-coated urea at 240 kg N ha^−1^, PCU). We found that water and N treatments had remarkable effects on the measured parameters in different plant tissues after transplanting, but the water and N interactions had insignificant effects. Tissue C:N, N:P and C:P ratios ranged from 14.6 to 52.1, 3.1 to 7.8, and 76.9 to 254.3 over the rice growing seasons, respectively. The root and stem-leaf C:N:P and panicle C:N ratios showed overall uptrends with a peak at harvest whereas the panicle N:P and C:P ratios decreased from filling to harvest. The AWD treatment did not affect the concentrations and accumulation of tissue C and N, but greatly decreased those of P, resulting in enhanced N:P and C:P ratios. N fertilization significantly increased tissue N concentration, slightly enhanced tissue P concentration, but did not affect tissue C concentration, leading to a significant increase in tissue N:P ratio but a decrease in C:N and C:P ratios. Our results suggested that the growth of rice in the Taihu Lake region was co-limited by N and P. These findings broadened our understanding of the responses of plant C:N:P stoichiometry to simultaneous water and N managements in subtropical high-yielding rice systems.

## Introduction

Nitrogen (N) is one of the most important mineral nutrients. It promotes large leaf area index [1], long duration of photosynthesis [2], high nutrient uptake [3], and ultimately high crop productivity [4], [5]. Along with N, phosphorous (P) is another vital mineral nutrient influencing plant photosynthesis assimilation and biomass production [2], [6], [7]. To meet the challenge of food security, a large amount of chemical fertilizers has been applied to the rice cropping systems, particularly in China [8]. Long-term use of high rates of fertilization with improper water and nutrient managements has resulted in low water and nutrient use efficiencies, leading to detrimental effects on ecology, environment and human health [4], [8]. The seasonal absorption, accumulation and allocation of N and P in rice may deserve special attention for implementing sound water and nutrient management practices in sustainable rice production systems.

Carbon (C), which provides the structural basis and constitutes a fairly stable 50% of a plant’s dry mass, can also act as a limiting element for plant [9]. Rice crop may play an important role in terrestrial C cycle by both C sequestrations through photosynthesis and C releases through residues decomposition and/or root respiration [10], [11]. Management practices such as irrigation and fertilization can influence crop physio-ecological activities [1], hence affect the sequestration and emission of C in paddy fields, which presumably have an effect on the mitigation of global warming and the stability of food security [12]. Most previous studies have focused on the accumulation and partitioning of dry matter of rice in response to elevated [CO_2_] (free-air CO_2_ enrichment) [5], [7], [13], [14]. However, little has been done to evaluate the effects of water and N managements on the assimilation, accumulation and distribution of C in rice plant.

Since C, N and P are strongly coupled in their biochemical functioning [9],[15] and their balance generally affects crop production and food-web dynamics [16], the C:N:P stoichiometry is the most investigated factor in ecological interactions. To date, C:N:P stoichiometry has been widely applied in diverse ecological processes, and successfully incorporated into explain many phenomena at all levels of biology, from genes and molecules to whole organisms and even to ecosystems and the biosphere [16]–[19]. Some measurements have already been made on the spatiotemporal variations, biological regulation mechanisms and the ecological implications of C:N:P stoichiometric ratios in soil, plant and litter at different trophic levels on a regional, national and even global scale [9], [17]–[20]. Elucidating changes in C:N:P ratios during plant growth could be useful in calibrating plant mechanistic models and developing terrestrial biogeochemical models [21], [22]. However, the seasonal changes of C:N:P ratios in rice plant, particularly with their responses to different water and N managements have not been well characterized.

In an earlier study, we investigated the effects of two controlled-release N fertilizers (CRNFs) (controlled-release bulk blending fertilizer and polymer-coated urea: BBF and PCU) under two irrigation regimes (continuous flooding and alternate wetting and drying: CF and AWD) in comparison with urea on the dry matter accumulation and partitioning, grain yield, and water and N use efficiencies in late-season rice in the Taihu Lake region of China, and found the agronomic performances played individually or jointly by the irrigation and N managements [4]. The objectives of this research were to: (1) investigate the seasonal changes of C, N and P concentrations, accumulation, allocation, and stoichiometric ratios in different plant tissues under different water and N managements, (2) get the relationships between tissue C:N:P ratios and rice grain yield, and (3) evaluate the limiting patterns of nutrients via C:N:P stoichiometry in rice production systems.

## Materials and Methods

(This work was unrelated to ethics issues, and no specific permit was required for the described field study, and we confirmed that the field study did not involve endangered or protected species).

### Site description

This study was conducted at Yuhang town of Zhejiang province, Taihu Lake region of China (30°21′50″ N, 119°53′17″ E) in 2010 and 2011. The study site has a subtropical monsoon climate with an average temperature of 16.2°C and an annual precipitation of 1290 mm. The soil type of the experimental field is hydromorphic paddy soil (Ferric-accumulic Stagnic Anthrosols). Initial soil properties of the plow layer (0–20 cm) were: pH 5.8 (1∶5, soil/water), soil organic C 21.75 g kg^−1^, total N 3.46 g kg^−1^, mineral N 24.04 mg kg^−1^, and total P 0.32 g kg^−1^. Single cropping of late-season rice has been widely adopted in the region. The average routine rate of fertilization is 240 kg N ha^−1^ (as urea, 46% N), 120 kg P_2_O_5_ ha^−1^ (as superphosphate, 12% P_2_O_5_), and 120 kg K_2_O ha^−1^ (as potassium chloride, 60% K_2_O) for one rice season.

### Experimental design

The field experiment was arranged in a split-plot design with three replications in both years. Main plots consisted of two irrigation regimes: CF and AWD. All plots were flooded during the first 10–14 days after transplanting (DAT), allowing seedlings to recover from the shock of transplanting prior to the imposition of AWD treatments. Further details on the application of CF and AWD were described by Ye et al. [4]. The daily temperature, rainfall, irrigation, and field water depth from transplanting to harvest under CF and AWD irrigation in 2010 and 2011 were also reported in detail in our previous publication [4]. Subplots consisted of four N treatments: the control (N0), a urea application of 240 kg N ha^−1^ (UREA), and two basal CRNF treatments both at the rate of 240 kg N ha^−1^ (BBF and PCU). The PCU (42% N) is one of the most widely used coated granular CRNF fertilizers, and the BBF (24% N-12% P_2_O_5_-12% K_2_O) is a compound CRNF product in which N source is made up of 70% controlled-release N and 30% ordinary quick-acting N. Both BBF and PCU (Kingenta Ecological Engineering Co., Ltd., Shandong, China) are 90 days of N release period and need only one-off fertilization.

Each plot was 6 m×3 m in size. All bunds were mulched with plastic film to minimize lateral seepage between adjacent plots. Individual inlet and outlet in the boundary side of the bunds were established in each plot for irrigation and drainage. A local late-season rice cultivar named Xiushui 134 (*Oryza sativa* L.) with high-yielding potential and pest resistance was used. Urea was applied in three splits for the late-season rice, 40% was basally applied (0 DAT), 40% was topdressed at the tillering stage (32 DAT), and the remaining 20% was topdressed at the panicle initiation stage (63 DAT). Full doses of superphosphate and potassium chloride in the N0, UERA and PCU treatments were applied basally at rates of 120 kg P_2_O_5_ ha^−1^ and 120 kg K_2_O ha^−1^, respectively. The 3-week-old seedlings were transplanted at a spacing of 25 cm×16 cm with three seedlings per hill on 23 June 2010 and 1 July 2011. Plots were regularly hand-weeded until canopy leaves were extremely crowded to prevent weed damage. The pests and diseases managements followed the local tradition. Final harvests were done on 9 November 2010 and 17 November 2011, and the growth duration was 140 days in all treatments for either year.

### Plant sampling and measurements

Five hills of plants (except border plants) in each plot were dug out by a shovel at seedling, tillering, booting, filling, and maturity stages (harvest), 1, 35, 56, 91, 140 DAT in both years. All visible root tissues were collected from the soil. Plants were washed free of soil and separated into two parts: root, stem-leaf before heading (75–80 DAT) and three parts: root, stem-leaf, and panicle after heading. All plant sub-samples were oven-dried to a constant weight at 70°C, weighted, and ground to sift through a 0.15 mm sieve for chemical measurements. Tissue C and N concentrations were determined with combustion technique on a Vario MAX CNS elemental analyzer (Elementar Analysensysteme GmbH, Hanau, Germany) [10]. Tissue P concentration was analyzed colorimetrically by the molybdenum blue method following digestion in concentrated H_2_SO_4_ and H_2_O_2_ [23].

### Calculations and statistical analysis

Accumulation of C, N and P in root, stem-leaf, and panicle at various growth stages was calculated from the element concentration multiplied by the dry matter. The element accumulation rate (kg ha^−1^ day^−1^) at each growth stage was obtained from the element accumulation divided by the number of days of the growth stage. Stoichiometric ratios of C:N, N:P and C:P in plant tissues were calculated on mass basis.

All statistical analyses were performed using PASW Statistics 18.0 (SPSS Inc. Chicago, USA). Combined analysis of variance using data from two years indicated that the interactions between years and irrigation regimes, and between years and N managements on the measured parameters (C, N and P concentrations, accumulation, partitioning, and stoichiometric ratios) were not significant in both seasons. Therefore, the data were arithmetic averaged across two years (a total of six replications) for further analyses [1]. Irrigation methods and N managements were considered as fixed factors. Two-way ANOVA were used to assess the effects of both water regimes (CF vs. AWD) and N managements (N0 vs. UREA vs. BBF vs. PCU) on the analysis of variance (*F*-value) of C, N and P concentrations, accumulation, partitioning, and C:N:P stoichiometric ratios in root, stem-leaf, and panicle at various growth stages, and also to test the interactions of water regimes × N managements. The least significant difference (LSD) test at the 0.05 probability level was used to compare significant differences among treatment means. Spearman rank correlation was performed to reveal the interrelations between C:N:P ratios of plant tissues and the grain yield at harvest.

## Results

Water and N managements did alter the patterns of C, N and P concentrations, accumulation and partitioning in different plant tissues after transplanting, but no significant water by N interaction effect was found at any stage of sampling (*P*>0.05). Besides, the effects of different N treatments on tissue C, N and P concentrations, accumulation and partitioning were always more significant than those of different irrigation regimes (Tables S1 and S2 in File S1). Because irrigation and N treatments had remarkable effects on the assimilation, accumulation and allocation of C, N and P in different organs, the plant C:N:P stoichiometry was greatly affected by the water and N managements, particularly during the late-cultivation period. However, the water × N interactions on tissue C:N:P ratios were generally not significant (except root N:P (*F* = 3.08, *P*<0.05) and stem-leaf N:P (*F* = 3.02, *P*<0.05) at tillering stage) through the rice growing seasons (Table S3 in File S1).

### C, N and P concentrations

**Figure 1:**
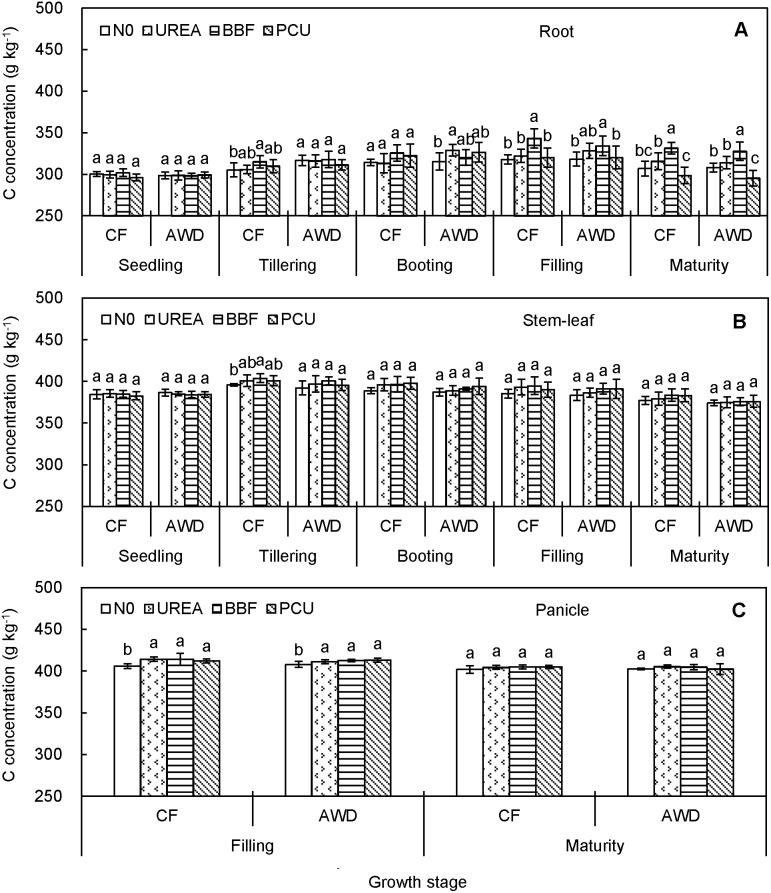
Seasonal changes of carbon concentration in root (A), stem-leaf (B), and panicle (C) of late-season rice under different water and N managements (2-year average). Vertical bars represent ± standard deviation of the mean (n = 6). The different letters listed above bars represent significant differences at *P*<0.05.

C concentration ranged from 295.3 to 343.2 g kg^−1^ in root, 374.1 to 403.7 g kg^−1^ in stem-leaf, and 401.9 to 414.2 g kg^−1^ in panicle over the rice growing seasons (Fig. 1). Root C concentration showed a remarkable increase from seedling (299.0 g kg^−1^) to filling (325.5 g kg^−1^), and then decreased to maturity (312.2 g kg^−1^). Stem-leaf C concentration increased only up to tillering (398.3 g kg^−1^), and then decreased to harvest (378.0 g kg^−1^). Panicle C concentration showed a slight decrease from filling (411.5 g kg^−1^) to maturity (403.9 g kg^−1^). Tissue C concentration was not obviously affected by the two irrigation regimes or by the three N-fertilized treatments, and UREA, BBF and PCU did not give consistently higher C concentrations than N0 from transplanting to harvest.

**Figure 2:**
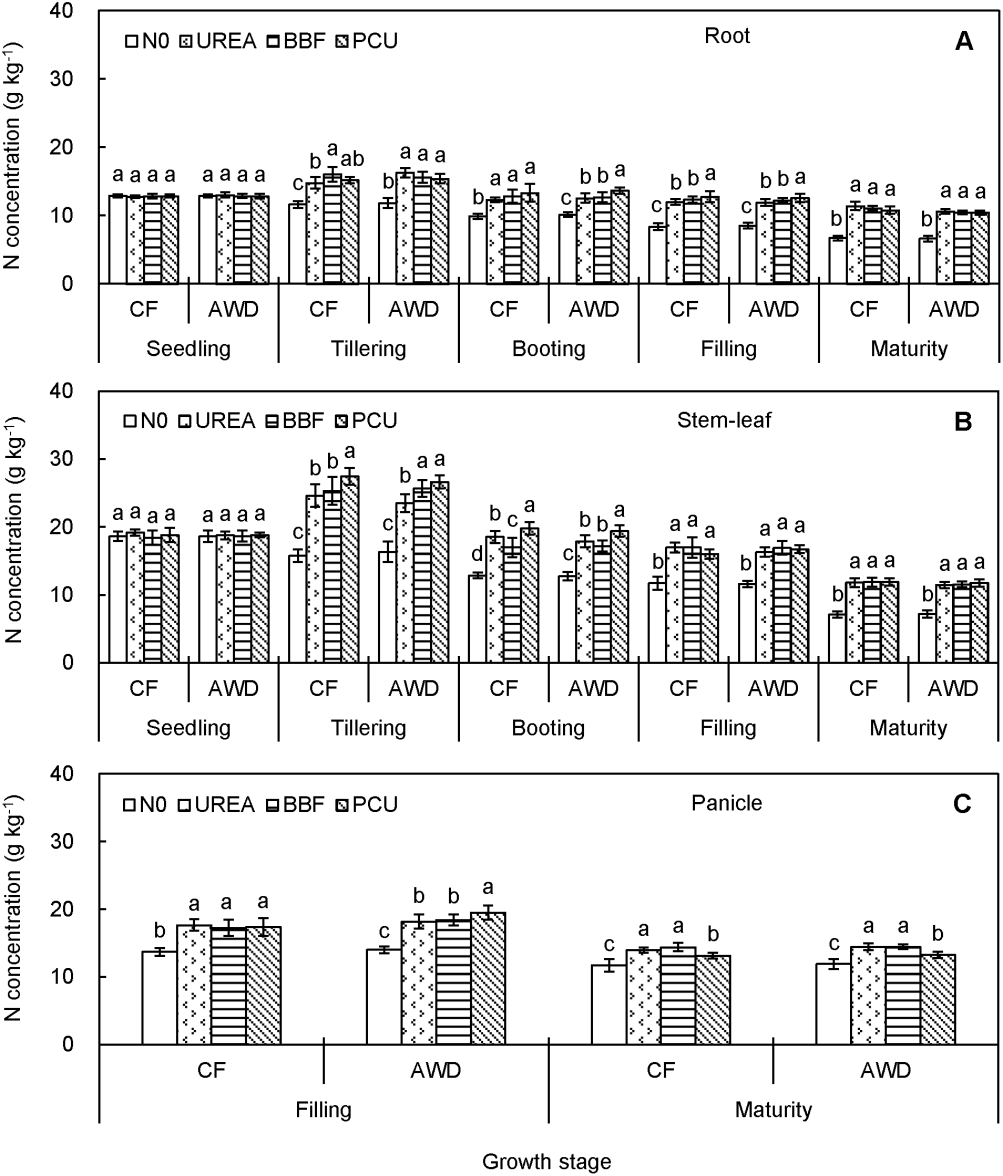
Seasonal changes of nitrogen concentration in root (A), stem-leaf (B), and panicle (C) of late-season rice under different water and N managements (2-year average). Vertical bars represent ± standard deviation of the mean (n = 6). The different letters listed above bars represent significant differences at *P*<0.05.

N concentration ranged from 6.6 to 16.3 g kg^−1^ in root, 7.1 to 27.4 g kg^−1^ in stem-leaf, and 11.7 to 19.5 g kg^−1^ in panicle over the planting seasons (Fig. 2). Both root and stem-leaf N concentrations in N0 treatment showed clearly decreasing trends from seedling (12.9 and 18.6 g kg^−1^) to harvest (6.7 and 7.2 g kg^−1^), while those in N-fertilized treatments first increased from seedling (12.9 and 18.8 g kg^−1^) to tillering (15.6 and 25.5 g kg^−1^) and then decreased to harvest (10.8 and 11.7 g kg^−1^). Panicle N concentration dropped from filling (17.0 g kg^−1^) to maturity (13.4 g kg^−1^) irrespective of N addition. There was no significant difference in tissue N concentration between CF and AWD at any stage of observation. As expected, N fertilization dramatically increased tissue N concentration after seedling, particularly during the late growth period. At harvest, N concentration was significantly increased by 64.7%, 61.2% and 59.0% in root, 62.6%, 63.9% and 65.7% in stem-leaf, and 20.4%, 22.2% and 11.9% in panicle in UREA, BBF and PCU compared with those of N0, respectively. However, no consistent significant differences among the three N-fertilized treatments were observed through the growing seasons.

**Figure 3:**
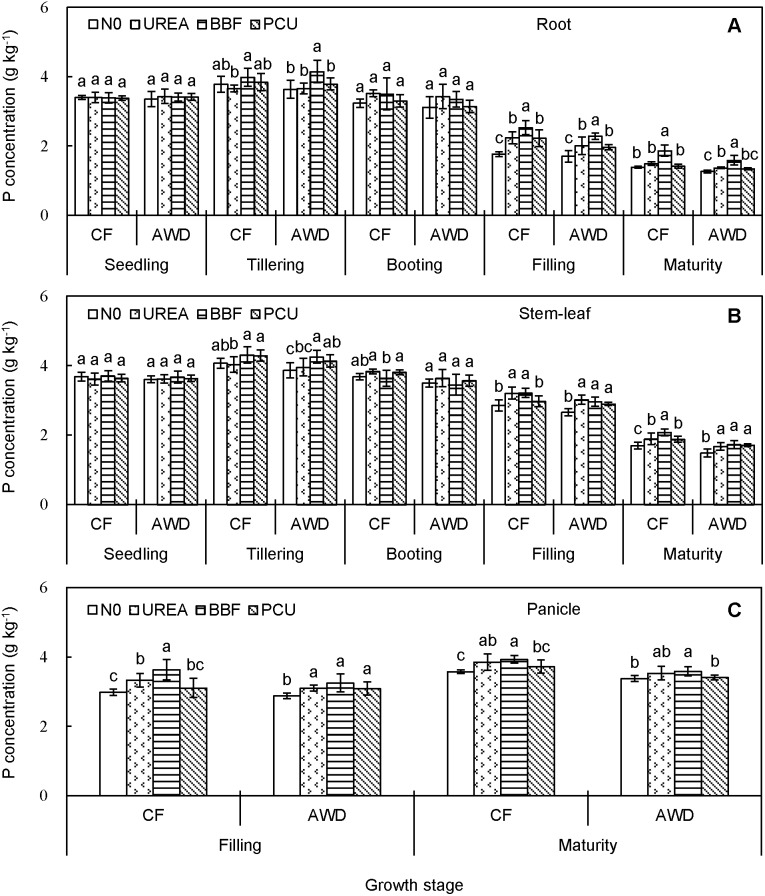
Seasonal changes of phosphorus concentration in root (A), stem-leaf (B), and panicle (C) of late-season rice under different water and N managements (2-year average). Vertical bars represent ± standard deviation of the mean (n = 6). The different letters listed above bars represent significant differences at *P*<0.05.

P concentration ranged from 1.3 to 4.2 g kg^−1^ in root, 1.5 to 4.3 g kg^−1^ in stem-leaf, and 2.9 to 3.9 g kg^−1^ in panicle over the planting seasons (Fig. 3). Both root and stem-leaf P concentrations displayed slight increases from seedling (3.4 and 3.6 g kg^−1^) to tillering (3.8 and 4.1 g kg^−1^), then gradual decreases until harvest (1.5 and 1.8 g kg^−1^). Interestingly, panicle P concentration increased from filling (3.2 g kg^−1^) to maturity (3.6 g kg^−1^), exhibiting an opposite trend to those of panicle C and N concentrations. Water regimes did alter tissue P concentration, particularly during the late growth period. Compared with CF, AWD significantly decreased P concentration by 9.1%, 5.8%, and 5.6% at filling and 9.6%, 12.5%, and 7.8% at maturity in root, stem-leaf, and panicle, respectively. Tissue P concentration was not obviously affected by the N fertilization before heading, hereafter enhanced by 5.7–38.6% at filling and 2.6–30.4% at maturity in those N-fertilized treatments compared with N0, though the differences among the four N treatments were not always significant at the *P*<0.05 level.

### C, N and P accumulation

**Figure 4:**
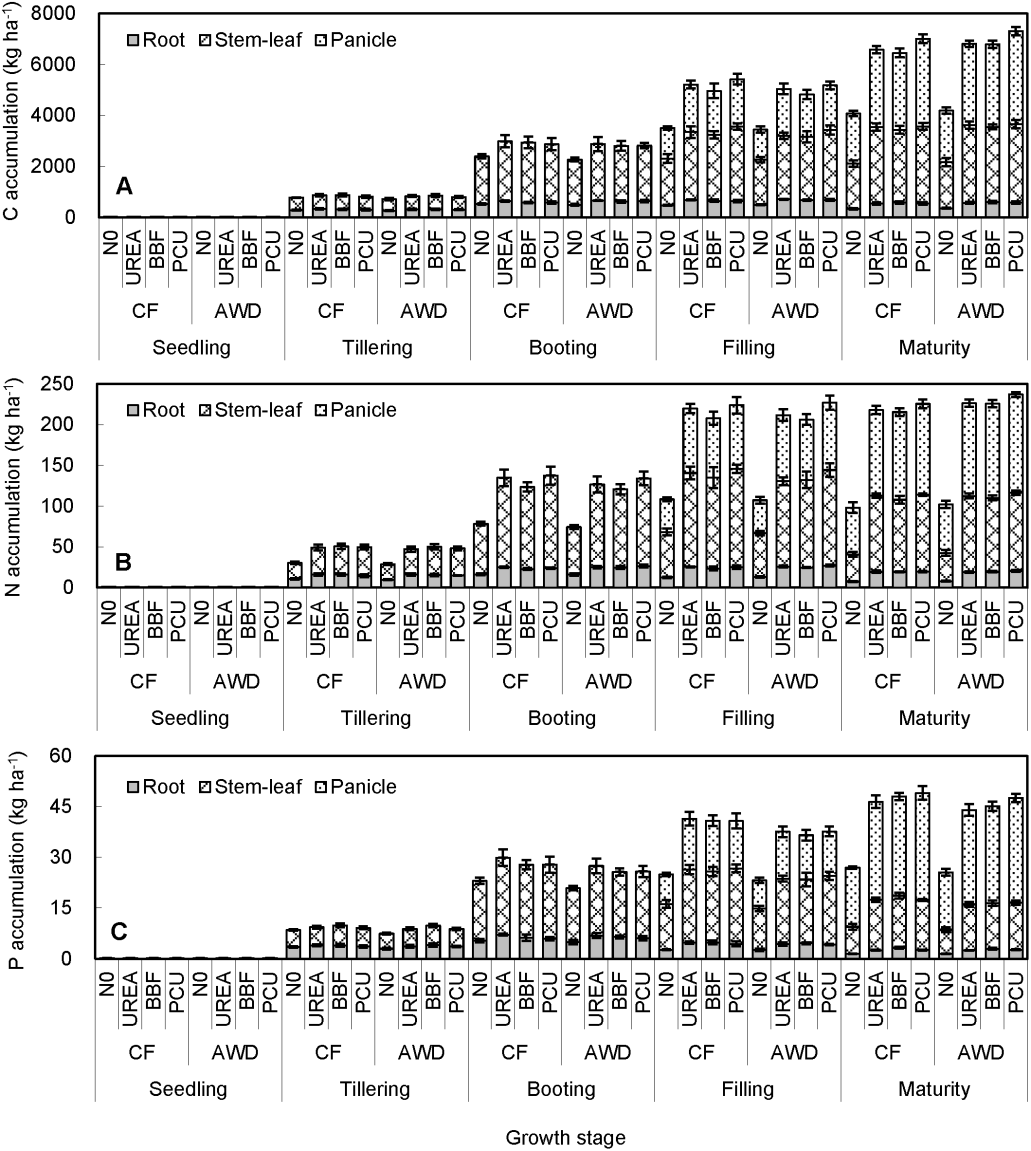
Seasonal changes of carbon (A), nitrogen (B) and phosphorus (C) accumulation in root, stem-leaf, and panicle of late-season rice under different water and N managements (2-year average). Vertical bars represent ± standard deviation of the mean (n = 6).

For the N0 control, both root and stem-leaf C accumulation peaked at booting stage (506 and 1824 kg ha^−1^). However, root C accumulation peaked at filling stage (656–695 kg ha^−1^) and stem-leaf C accumulation peaked at harvest (2904–3052 kg ha^−1^) for the N-fertilized treatments (Fig. 4A). Panicle C accumulation peaked at harvest irrespective of N-fertilizer application (1996, 3120, 3132, and 3548 kg ha^−1^ in N0, UREA, BBF, and PCU, respectively). The maximum rates of average C accumulation across all treatments were observed at booting in root and stem-leaf (13.8 and 78.1 kg ha^−1^ day^−1^) and at filling in panicle (46.8 kg ha^−1^ day^−1^). Tissue C accumulation did not differ remarkably between CF and AWD or among UREA, BBF and PCU at any stage of sampling, but increased significantly in the N-fertilized treatments compared with N0 after tillering. At harvest, C accumulation was enhanced by 59.1%, 71.3% and 62.5% in root, 69.5%, 62.1% and 70.4% in stem-leaf, and 56.3%, 56.9% and 77.8% in panicle in UREA, BBF and PCU compared with those of N0, respectively.

The maximum N accumulation in root and stem-leaf was observed at booting in N0 control (16.1 and 60.2 kg ha^−1^), but at filling in treatments with N addition (25.5, 24.0 and 26.0 kg ha^−1^ for root and 109.6, 109.5 and 118.9 kg ha^−1^ for stem-leaf in UREA, BBF and PCU, respectively) (Fig. 4B). Panicle N accumulation showed a clear uptrend from filling (68.4 kg ha^−1^) to maturity (98.9 kg ha^−1^) regardless of N addition. The maximum rates of average N accumulation across all treatments were observed at tillering in root (0.39 kg ha^−1^ day^−1^), booting in stem-leaf (3.03 kg ha^−1^ day^−1^), and filling in panicle (1.96 kg ha^−1^ day^−1^). There was no significant difference in tissue N accumulation between CF and AWD at all observation stages. Obviously, N addition greatly improved plant N uptake after seedling, and the difference between fertilized and unfertilized plots expanded continuously with crop growth. At harvest, N accumulation was enhanced by 155.8%, 157.5% and 167.4% in root, 170.4%, 162.8% and 179.1% in stem-leaf, and 109.4%, 111.5% and 116.0% in panicle in UREA, BBF and PCU compared with those of N0, respectively. Besides, PCU obtained higher plant N accumulation than BBF and UREA after tillering, suggesting that PCU tended to be more effective in promoting the N transformation from soil to plant during rice growth period.

P accumulation increased remarkably up to booting in both root and stem-leaf (6.1 and 20.0 kg ha^−1^), and up to harvest in panicle (26.5 kg ha^−1^) (Fig. 4C). The maximum rates of average P accumulation across all treatments were observed at booting in root and stem-leaf (0.12 and 0.70 kg ha^−1^ day^−1^) and at filling in panicle (0.36 kg ha^−1^ day^−1^). AWD decreased tissue P accumulation after seedling, particularly at booting and filling stages (*P*<0.05). N fertilization had a positive effect on plant P uptake which increased with the increase of plant maturity. At harvest, P accumulation was significantly enhanced by 67.9%, 108.3% and 75.6% in root, 89.2%, 91.7% and 90.5% in stem-leaf, and 64.9%, 68.3% and 81.6% in panicle in UREA, BBF and PCU compared with those of N0, respectively. However, no consistent significant differences among the three N-fertilized treatments were observed through the whole rice seasons.

### C, N and P partitioning

Crop element composition depends on the process of accumulation, translocation and allocation of the elements. The seasonal variations of tissue C, N and P partitioning were shown in Table 1. C partitioning displayed a sharp decrease from transplanting (37.6%) to harvest (8.3%) in root but a remarkable increase from filling (34.8%) to maturity (47.9%) in panicle. Stem-leaf C partitioning initially increased up to booting (78.5%) and then decreased with the increase of plant maturity (43.8% at harvest). Tissue C partitioning was comparable between the two irrigation treatments at seedling, tillering and maturity stages, but increased significantly in root (7.7% and 7.9%) vs. decreased in stem-leaf (2.0% and 2.1%) in AWD compared with CF at booting and filling stages. No significant differences in tissue C partitioning among the four N treatments were found before ripening (110–130 DAT), while the highest C partitioning of root, stem-leaf, and panicle was observed in BBF (8.9%), UREA (45.3%), and PCU (49.5%) at harvest, respectively.

**Table 1:** Seasonal changes of carbon, nitrogen and phosphorus partitioning in root, stem-leaf, and panicle of late-season rice under different water and N managements (2-year average).

Growth stage	Water regime	Nitrogen treatment	C partitioning (%)			N partitioning (%)			P partitioning (%)		
			Root	Stem-leaf	Panicle	Root	Stem-leaf	Panicle	Root	Stem-leaf	Panicle
Seedling	CF	N0	38.0 a	62.0 a	-	35.2 a	64.8 a	-	42.2 a	57.8 a	-
		UREA	37.1 a	62.9 a	-	33.6 a	66.4 a	-	42.0 a	58.0 a	-
		BBF	36.3 a	63.7 a	-	33.7 a	66.3 a	-	40.1 a	59.9 a	-
		PCU	38.5 a	61.5 a	-	35.7 a	64.3 a	-	43.0 a	57.0 a	-
		Avg.	37.5 A	62.5 A	-	34.6 A	65.4 A	-	41.7 A	58.3 A	-
	AWD	N0	37.3 a	62.7 a	-	34.8 a	65.2 a	-	41.8 a	58.2 a	-
		UREA	37.2 a	62.8 a	-	34.7 a	65.3 a	-	42.0 a	58.0 a	-
		BBF	37.9 a	62.1 a	-	35.1 a	64.9 a	-	42.2 a	57.8 a	-
		PCU	38.7 a	61.3 a	-	35.6 a	64.4 a	-	43.3 a	56.7 a	-
		Avg.	37.8 A	62.2 A	-	35.1 A	64.9 A	-	42.3 A	57.7 A	-
Tillering	CF	N0	36.4 a	63.6 a	-	35.5 a	64.5 c		40.9 a	59.1 a	
		UREA	37.9 a	62.1 a	-	32.5 b	67.5 b	-	42.0 a	58.0 a	-
		BBF	35.9 a	64.1 a	-	31.3 bc	68.7 ab	-	39.9 a	60.1 a	-
		PCU	36.4 a	63.6 a	-	29.1 c	70.9 a	-	39.8 a	60.2 a	-
		Avg.	36.6 A	63.4 A	-	32.1 A	67.9 A	-	40.7 A	59.3 A	-
	AWD	N0	36.3 a	63.7 a	-	33.8 a	66.2 b	-	39.9 a	60.1 a	-
		UREA	37.5 a	62.5 a	-	34.4 a	65.6 b	-	41.1 a	58.9 a	-
		BBF	36.4 a	63.6 a	-	30.5 b	69.5 a	-	41.3 a	58.7 a	-
		PCU	37.4 a	62.6 a	-	30.5 b	69.5 a	-	41.1 a	58.9 a	-
		Avg.	36.9 A	63.1 A	-	32.3 A	67.7 A	-	40.8 A	59.2 A	-
Booting	CF	N0	21.6 a	78.4 a	-	20.8 a	79.2 b	-	23.1 a	76.9 a	-
		UREA	21.4 a	78.6 a	-	18.6 b	81.4 a		24.0 a	76.0 a	
		BBF	19.6 a	80.4 a	-	18.3 b	81.7 a	-	22.3 a	77.7 a	-
		PCU	20.3 a	79.7 a	-	17.5 b	82.5 a	-	21.5 a	78.5 a	-
		Avg.	20.7 B	79.3 A	-	18.8 B	81.2 A	-	22.7 B	77.3 A	-
	AWD	N0	21.8 a	78.2 a	-	21.4 a	78.6 a	-	23.4 a	76.6 a	-
		UREA	22.9 a	77.1 a	-	19.8 a	80.2 a	-	24.9 a	75.1 a	-
		BBF	22.1 a	77.9 a	-	20.4 a	79.6 a	-	25.2 a	74.8 a	-
		PCU	22.4 a	77.6 a	-	19.7 a	80.3 a	-	23.5 a	76.5 a	-
		Avg.	22.3 A	77.7 B	-	20.3 A	79.7 B	-	24.3 A	75.7 B	-
Filling	CF	N0	13.6 a	52.3 a	34.1 a	11.6 a	51.3 a	37.1 a	10.6 b	54.3 a	35.1 a
		UREA	13.1 a	51.1 a	35.8 a	11.6 a	52.8 a	35.6 a	11.5 a	52.3 ab	36.2 a
		BBF	13.2 a	52.3 a	34.7 a	11.2 a	53.7 a	35.1 a	11.7 a	51.6 b	36.7 a
		PCU	11.6 b	54.1 a	34.3 a	11.2 a	53.9 a	34.9 a	10.8 b	54.9 a	34.3 a
		Avg.	12.8 B	52.5 A	34.7 A	11.4 B	52.9 A	35.7 A	11.1 A	53.2 A	35.7 A
	AWD	N0	14.2 a	51.4 ab	34.4 a	12.1 a	50.3 a	37.6 a	11.2 b	52.8 a	36.0 a
		UREA	14.1 a	49.4 b	36.5 a	12.2 a	49.6 a	38.2 a	11.6 b	51.5 a	36.9 a
		BBF	14.0 a	51.5 ab	34.5 a	11.9 a	52.1 a	36.0 a	12.6 a	51.5 a	35.9 a
		PCU	13.2 a	52.9 a	33.9 a	11.8 a	51.7 a	36.5 a	11.2 b	53.9 a	34.9 a
		Avg.	13.9 A	51.3 B	34.8 A	12.0 A	50.9 B	37.1 A	11.6 A	52.5 A	35.9 A
Maturity	CF	N0	8.2 b	43.4 b	48.4 a	7.4 b	34.2 c	58.4 a	5.6 b	29.4 b	65.0 a
		UREA	8.1 b	45.7 a	46.2 b	8.9 a	42.9 a	48.2 b	5.4 b	32.2 a	62.4 b
		BBF	9.0 a	44.2 b	46.8 b	8.9 a	41.1 b	50.0 b	6.7 a	32.2 a	61.1 b
		PCU	7.7 b	43.3 b	49.0 a	8.7 a	41.9 ab	49.4 b	5.3 b	30.3 ab	64.4 a
		Avg.	8.3 A	44.1 A	47.6 A	8.5 A	40.0 A	51.5 A	5.8 A	31.0 A	63.2 B
	AWD	N0	8.5 b	43.2 b	48.3 b	7.5 b	34.0 b	58.5 a	5.7 b	27.9 b	66.4 a
		UREA	8.3 b	44.8 a	46.9 c	8.4 a	41.3 a	50.3 b	5.6 b	31.0 a	63.4 b
		BBF	8.9 a	43.4 b	47.7 bc	8.6 a	40.2 a	51.2 b	6.5 a	29.9 a	63.6 b
		PCU	7.9 c	42.1 c	50.0 a	8.6 a	40.5 a	50.9 b	5.5 b	29.3 ab	65.2 ab
		Avg.	8.4 A	43.4 A	48.2 A	8.3 A	39.0 A	52.7 A	5.8 A	29.6 B	64.6 A

Within a column for each growth stage, means followed by the same letter are not significantly different at *P*<0.05 by LSD test. Lowercase and uppercase letters indicate comparisons among four N treatments and between two irrigation regimes, respectively.

Tissue N partitioning exhibited a similar seasonal pattern to C partitioning (Table 1). N partitioning was also significantly higher in root (8.1% and 5.3%) but lower in stem-leaf (2.0% and 3.9%) in AWD than CF at booting and filling stages. There were no clear differences in tissue N partitioning among the four N treatments before ripening. However, N0 resulted in the lowest N partitioning in root (7.5%) and stem-leaf (34.1%) but the highest one in panicle (58.4%) at harvest.

The seasonal changes of P partitioning in root, stem-leaf, and panicle were similar to those of C and N partitioning (Table 1). Tissue P partitioning was unaffected by the two water regimes at seedling, tillering and filling stages, but increased significantly by 6.9% in root at booting and 2.2% in panicle in AWD compared with CF at harvest. Tissue P partitioning was comparable among the four N treatments at all observation stages, indicating that N fertilization had no significant influence on P allocation.

### C:N:P stoichiometric ratios

**Figure 5:**
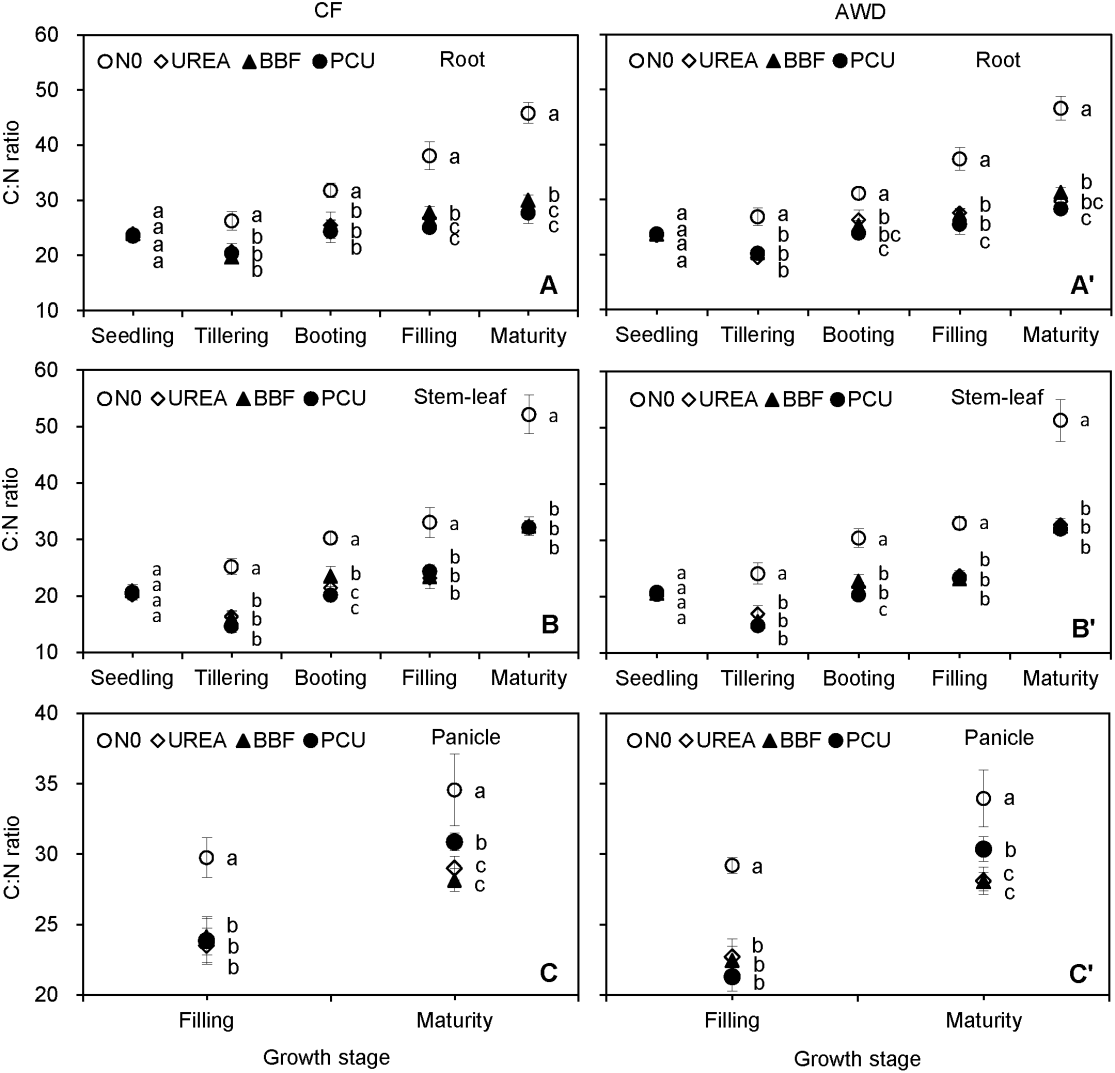
Seasonal changes of C:N ratio in root (A, A’), stem-leaf (B, B’), and panicle (C, C’) of late-season rice under different water and N managements (2-year average). Vertical bars represent ± standard deviation of the mean (n = 6). The different letters listed around bars represent significant differences at *P*<0.05.

C:N ratio ranged from 19.4 to 46.6 in root, 14.6 to 52.1 in stem-leaf, and 21.3 to 34.6 in panicle over the planting seasons (Fig. 5). Tissue C:N ratio increased gradually with crop growth after seedling, and peaked at harvest. Notably, C:N ratio was comparable between root and stem-leaf during the rice seasons, though C and N concentrations were much higher in stem-leaf than root (Figs. 1 and 2). Tissue C:N ratio did not differ significantly between CF and AWD or among UREA, BBF and PCU at any stage of observation, but decreased significantly by 23.4–40.1%, 17.8–33.3%, 21.0–32.9%, and 10.6–39.3% in the N-fertilized treatments compared with those of N0 at tillering, booting, filling, and maturity stages, respectively.

**Figure 6:**
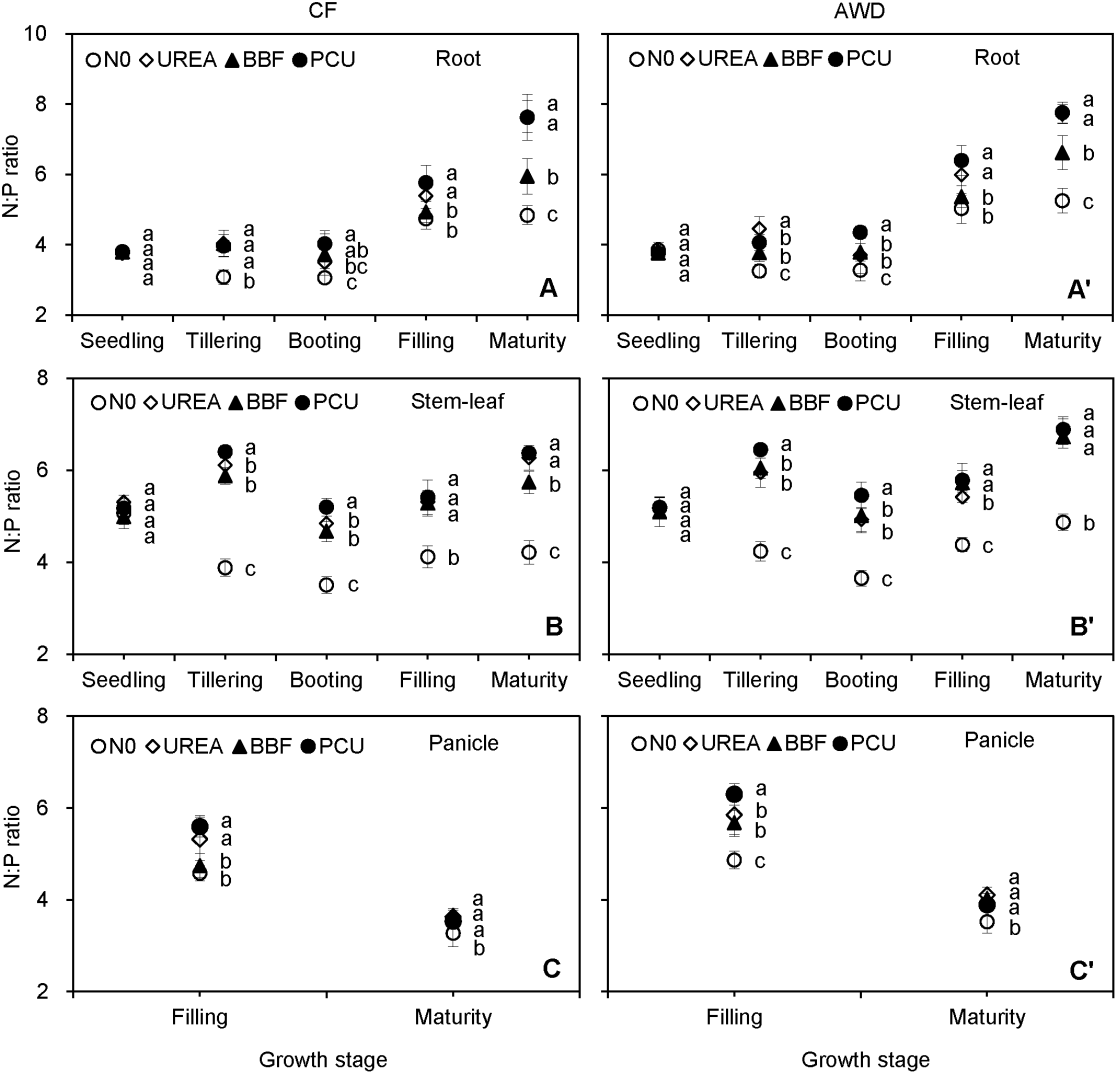
Seasonal changes of N:P ratio in root (A, A’), stem-leaf (B, B’), and panicle (C, C’) of late-season rice under different water and N managements (2-year average). Vertical bars represent ± standard deviation of the mean (n = 6). The different letters listed around bars represent significant differences at *P*<0.05.

N:P ratio ranged from 3.1 to 7.8 in root, 3.5 to 6.9 in stem-leaf, and 3.3 to 6.3 in panicle over the planting seasons (Fig. 6). Root N:P ratio showed no obvious difference from seedling (3.8) to tillering (3.7), then increased remarkably up to maturity (6.7). Stem-leaf N:P ratio exhibited a slight increase from booting (4.7) to maturity (6.0). Panicle N:P ratio displayed a 31.0% average reduction from filling (5.4) to maturity (3.7). The N:P ratio of panicle was significantly lower than those of root and stem-leaf at harvest (81.0% and 62.1%), though N and P concentrations of panicle were the highest among the three plant parts (Figs. 2 and 3). Tissue N:P ratio was comparable between the two irrigation regimes before heading, but enhanced significantly by 9.5% and 4.9% in root, 5.8% and 12.1% in stem-leaf, and 12.1% and 10.2% in panicle in AWD compared with CF at filling and maturity stages, respectively. N fertilization significantly increased tissue N:P ratio after seedling. The average N:P ratio was 52.2%, 24.6% and 52.5% higher in root, 44.6%, 37.3% and 46.0% higher in stem-leaf, and 13.7%, 13.2% and 9.2% higher in panicle in UREA, BBF and PCU than those of N0 at harvest, respectively. No consistent significant differences in N:P ratios of stem-leaf and panicle among the three N-fertilized treatments were observed through the rice growing seasons, except that UREA and PCU obtained significantly greater root N:P ratios (22.2% and 22.4%) than BBF at harvest.

**Figure 7:**
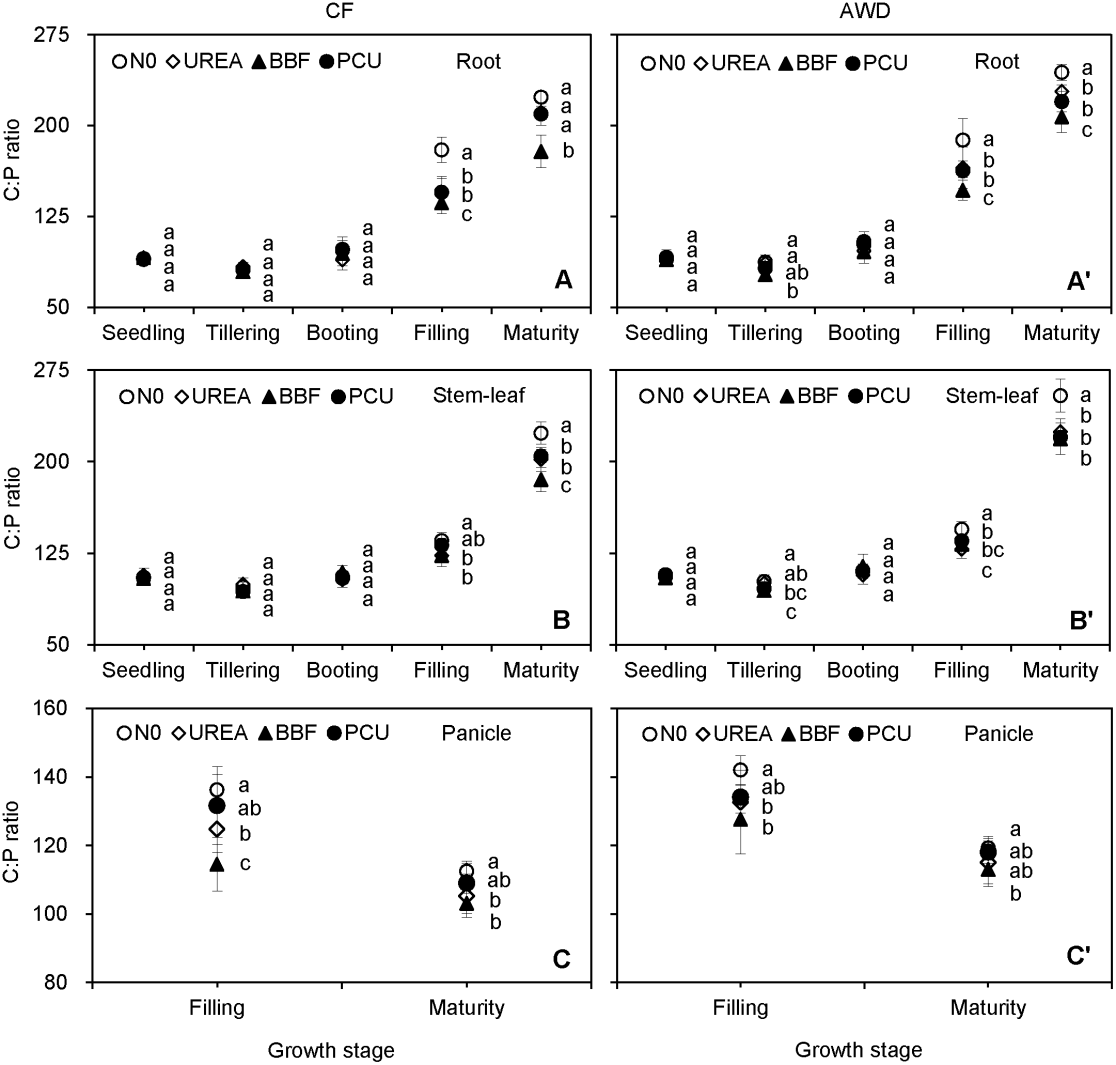
Seasonal changes of C:P ratio in root (A, A’), stem-leaf (B, B’), and panicle (C, C’) of late-season rice under different water and N managements (2-year average). Vertical bars represent ± standard deviation of the mean (n = 6). The different letters listed around bars represent significant differences at *P*<0.05.

C:P ratio ranged from 76.9 to 244.1 in root, 94.0 to 254.3 in stem-leaf, and 103.1 to 142.0 in panicle over the growing seasons (Fig. 7). Both root and stem-leaf C:P ratios fluctuated at around 100 before booting and then increased to 215.4 and 216.6 at harvest. As with panicle N:P ratio, panicle C:P ratio also decreased from filling (130.7) to maturity (111.9). The C:P ratio of panicle was significantly lower than those of root and stem-leaf at harvest (92.5% and 93.6%), though panicle possessed the highest C and P concentrations (Figs. 1 and 3). Tissue C:P ratio did not change significantly in response to irrigation nor to N fertilization before heading, but decreased by 5.3–9.4% at filling and 8.4–12.6% at maturity in CF compared with AWD, and reduced by 3.8–28.6% at filling and 2.0–17.1% at maturity in the N-fertilized treatments compared with N0 (although not in all cases significantly).

### Correlation studies

Tissue C:N ratio displayed significantly positive correlation with tissue C:P ratio but negative correlation with tissue N:P ratio (Table 2). Meanwhile, C:N ratios of root, stem-leaf, and panicle were significantly and positively correlated with each other. Similar patterns of correlation were also found for N:P and C:P ratios. These results indicated that C:N:P stoichiometric ratios were highly correlated both within and across plant tissues.

**Table 2:** Spearman rank correlations among tissue C:N:P stoichiometric ratios and grain yield of late-season rice at harvest.

Trait^a^	*C:N*_R_	*C:N*_SL_	*C:N*_P_	*N:P*_R_	*N:P*_SL_	*N:P*_P_	*C:P*_R_	*C:P*_SL_	*C:P*_P_	*GY*
*C:N*_R_	1									
*C:N*_SL_	**0.648** **	1								
*C:N*_P_	**0.336** *	**0.509** **	1							
*N:P*_R_	**−0.870** **	**−0.618** **	**−0.390** **	1						
*N:P*_SL_	**−0517** **	**−0.559** **	**−0.532** **	**0.667** **	1					
*N:P*_P_	**−0.315** *	**−0.332** *	**−0.661** **	**0.385** **	**0.677** **	1				
*C:P*_R_	**0.327** *	**0.365** *	**0.366** *	0.031^ns^	**−**0.073^ns^	**−**0.010^ns^	1			
*C:P*_SL_	**0.494** **	**0.605** **	**0.374** **	**−**0.281^ns^	0.076^ns^	0.072^ns^	**0.637** **	1		
*C:P*_P_	**0.306** *	**0.302** *	**0.307** *	**−**0.114^ns^	0.163^ns^	**0.437** **	**0.504** **	**0.651** **	1	
*GY*	**−0.699** **	**−0.633** **	**−0.302** *	**0.852** **	**0.832** **	**0.458** **	0.200^ns^	**−**0.158^ns^	0.094^ns^	1

Data from both CF and AWD treatments in both years were included (n = 48).

a *C:N_i_*, *N:P_i_* and *C:P_i_*: C:N, N:P and C:P ratios in different plant tissues, *i* refers to root (R), stem-leaf (SL), and panicle (P), respectively. *GY*: grain yield (data was drawn from Ye et al. [4]).

*Significant at *P*<0.05.

**Significant at *P*<0.01.

ns No significant.

Tissue N:P ratio was significantly and positively correlated with grain yield, while tissue C:N ratio got the opposite correlations. No obvious correlations were found between tissue C:P ratio and grain yield. Herein, tissue C:N and N:P ratios could have much more important implications than C:P ratio for expressing ecological stoichiometric relations in rice crop.

## Discussion

### Seasonal changes of C, N and P concentrations under different water and N managements

The C, N and P concentrations differed remarkably with plant tissues and crop growth stages. The overall increases of N and P concentrations in root and stem-leaf from seedling to tillering likely reflected the rapid soil exploration and high nutrient uptake by the crop roots. Besides, N and P concentrations in root increased only up to tillering while C concentration increased up to filling (Fig. 1), indicating that root C concentration was less affected by crop senescence during the late growth period. For stem-leaf, there exhibited systematic reductions in C, N and P concentrations from tillering to harvest (Figs. 1, 2 and 3). Decreases of nutrient concentration in vegetative parts with ontogenetic development of individual plants were documented not only in rice [5], [7], but also in corn, wheat, barley, and soybean [3], [24], resulting from an analogous dilution effect caused by increased plant size and biomass [13], [25], [26], [27]. Notably, P concentration in panicle increased from filling to maturity (Figs. 1, 2 and 3), showing an opposite trend to those of C and N concentrations. This could be explained by the fact that rapidly growing organ needs relatively more P-rich ribosomal RNA (approx. 9% by mass) to maintain rapid rate of protein synthesis [28], [29].

We noted different responses of C, N and P concentrations to CF and AWD in different plant tissues and at different growth stages, confirming that water availability played a key role in mediating element status in rice plant. Tissue C and N concentrations were comparable between CF and AWD (Figs. 1 and 2), implying that the water-saving irrigation had only a marginal effect on crop C and N assimilation. However, tissue P concentration was significantly reduced by AWD after heading (Fig. 3), probably because soil drying increased soil P sorption and led to less P availability for plant [30]. These results implied that the process governing tissue P concentration might be independent of those determining C and N concentrations. Indeed, in terms of plant, P is mainly derived from soil whereas C and N have diversified sources (e.g. CO_2_ enrichment, SOC mineralization, N deposition and N fixation). Therefore, soil water conditions could have more influence on P compared with C and N.

Tissue C, N and P concentrations responded differently to N fertilization. C concentration was almost unaffected by N fertilization (Fig. 1), in line with previous researches [18], [31], mainly due to the stable plant carbon composition and structural basis [9]. N and P concentrations were positively affected by the N application for most stages after tillering (Figs. 2 and 3), consistent with the reports of Bélanger et al. [25] who stated that increases in N concentration from increased N fertilization resulted in increased P concentration. This phenomenon was partly attributed to the increased capacity of roots to absorb more nutrients [24], and partly because of the stimulated P mobilization by the enhanced extracellular phosphatase activity [6], [32] and/or mycorrhizal colonization [27], [33].

### Seasonal changes of C, N and P accumulation and partitioning under different water and N managements

An unsynchronized tissue C, N and P accumulation was observed during rice growth. The highest accumulation of C, N and P in root, stem-leaf, and panicle emerged at different growth stages. N and P uptakes in root and stem-leaf were much lower at maturity than those at filling or even booting stage, while C, N and P accumulation in panicle all peaked at final harvest (Fig. 4). These results illustrated that carbohydrate provided by photosynthesis and nutrient derived by N and P remobilization were translocated from the senesced vegetative parts to the newly generated reproductive parts during the reproductive period [3]. The different behaviors among plant C, N and P accumulation were possibly due to the high flexibility of element composition and migration as a result of the trade-off between elements uptake and storage efficiency by the plant [34].

The complete knowledge of element allocation among plant organs is critical to evolutionary explanations of plant functional diversity [35]. As for the C, N and P partitioning of late-season rice in this study (Table 1), seasonal decreasing trends in root vs. increasing trends in panicle were exhibited. Stem-leaf was more like a transmission part between root and panicle, as its element partitioning initially increased during the early vegetative period and then decreased during the late reproductive period. Such seasonal allocation patterns of C, N and P were closely linked to (1) the obvious remobilization and retranslocation of C, N and P from root to aboveground parts before heading, and from stem-leaf to panicle hereafter, (2) the corresponding seasonal changes of biomass partitioning in different organs [4], and (3) the combined changes in plant nutrient absorbability, leaf phenology, and soil nutrient availability [32], [33]. Besides, C, N and P proportions in panicle increased substantially from 34.8%, 36.5% and 35.7% at filling to 47.9%, 52.1% and 63.9% at maturity (Table 1). The rapid increases in panicle N and P fractions were in line with the data reported by Yang et al. [5], [7] who found that the average N and P proportions were 10% and 7% at heading and 61% and 56% at maturity in panicle to the total aboveground rice plant. A similar pattern of element allocation was also found in maize [2] and wheat [3], demonstrating that the grain rather than stem-leaf was the major and most active sink for assimilated C, N and P in field crops. Furthermore, the reproductive allocation of N (48.2–58.5%) and P (61.1–66.4%) to the panicle exceeded that of C (46.2–48.4%) at harvest (Table 1), implying that the grain reproduction required higher fractions of N and P than C in rice.

Since intermittent irrigation has certain influence on the microclimatic conditions of paddy fields [1], [36], AWD would affect crop C assimilation and nutrient uptake. In fact, plant C and N accumulation was not remarkably affected by irrigation regimes through the rice growing seasons (Figs. 4A and 4B), whereas plant P accumulation was decreased by AWD compared with CF after seedling (Fig. 4C), resulting from the decreased available P in soil under the intermittent irrigation. This phenomenon implied that the AWD irrigation influenced P accumulation in a different way from its effect on the C and N accumulation. Water regimes affected tissue C, N and P partitioning after tillering (Table 1). In general, AWD obtained lower proportions of C, N and P in stem-leaf but higher ones in root and panicle (although not in all cases significantly) especially where N supply was abundant, mainly due to the functional shifts of plant organs and the distributional differences of biomass and nutrients [33] in response to the water-saving irrigation.

N fertilization greatly enhanced tissue C, N and P accumulation after transplanting (Fig. 4), resulting from both increased tissue element concentration (Figs. 1, 2 and 3) and biomass [4]. Meanwhile, N fertilizer (especially the PCU) application delayed the emerging of peak values of C and N accumulation in root and stem-leaf, owing to the extended soil N availability. In addition, the maximum rates of average C, N and P accumulation always appeared at the booting stage, mostly attributing to the fact that fast-growing plant tissues need relatively more elements to support rapid rate of cell proliferation. In order to take full advantage of the high uptake rate of N during the middle growth period and facilitate N harvesting during the late growth period, the delay of N release was necessary, which could be realized by the CRNFs [5]. In fact, the PCU enhanced not only N uptake but also C and P accumulation in panicle, shoot and whole plant compared with BBF and UREA at an equivalent N rate (Fig. 4). As a compound CRNF product, BBF failed to achieve a comparative effect on the increment of element accumulation as the PCU, owing to the lower components of controlled-release N source (70%) and the less effectiveness in controlling N release than the PCU [4]. N fertilization also altered N partitioning in different plant tissues. Zero N addition resulted in the lowest N partitioning in root (7.5%) and stem-leaf (34.1%) but the highest one in panicle (58.4%) at harvest (Table 1), which was in agreement with Yang et al. [5], suggesting that rice has evolved some internal regulation and conveyance strategies [37] to promote the absorbed N preferentially transferred from vegetative part to the reproductive organ when confronted with soil N deficiency.

### Seasonal variations of C:N:P ratios in different tissues under different water and N managements

C:N:P stoichiometry is an important and sensitive index reflecting diverse ecological processes. Knowledge on the C:N ratio of crop residues is of great importance for modelling C and N dynamics in agricultural systems [38]. We found that the stem-leaf C:N ratio showed an overall uptrend after seedling (Fig. 5), agreeing with the results of Ruan et al. [39] who observed that leaf C:N ratio increased from 20.8 at heading to 30.6 at filling and then to 32.0 at maturity in hybrid rice varieties. The stem-leaf C:P ratio exhibited a similar increasing trend as stem-leaf C:N ratio, owing to the reduced allocation of N and P to the senesced leaves. Panicle, which had higher C, N and P concentrations than root and stem-leaf, presented the lowest C:N, N:P and C:P ratios at harvest (Figs. 5, 6 and 7). Gan et al. [38] found that C:N ratio of seed (6–17) was significantly lower than those of straw (14–55) and root (17–75) at maturity in oilseed and pulse crops. Bélanger et al. [25] reported that grain N:P ratio (2.6–7.4) was lower and less variable than those of whole shoot (3.6–12.9) and the uppermost collared leaf (6.8–16.6) in maize. These results revealed that the storage-related tissues (panicle/seed) which optimized N and P use relative to C assimilation for grain production possessed lower C:N:P ratios than the growth-related tissues (stem/leaf).

Water availability that influences leaf phenology and photosynthesis rate could affect plant growth, nutritional status, and ultimately the stoichiometric ratios [32]. In this study, AWD irrigation did not significantly affect the tissue C:N ratio (Fig. 5), because neither tissue C nor N concentrations varied significantly under water-saving irrigation (Figs. 1 and 2). Besides, rice was unlikely to be water-limited under current experimental conditions because the AWD irrigation experienced in this study was within the ‘Safe AWD’ threshold (water level at 150 mm below the soil surface), and the soil in AWD plots was kept relatively wet and saturated during the non-submerged periods [4]. Plant N:P and C:P ratios were significantly enhanced by AWD irrigation at filling and maturity stages (Figs. 6 and 7), resulting from the reduced tissue P concentration under the alternate submergence-nonsubmergence (Fig. 3). These results indicated that the C:N ratio was much less sensitive to water conditions than the other two stoichiometric ratios, and confirmed that the AWD irrigation had important implications for plant-mediated C, N and P biogeochemical cycling in rice production systems. Lü et al. [32] noted that when water availability was enhanced, higher tissue P and unaltered N concentrations resulted in lower foliar N:P ratio in grassland plant species. Sardans & Peñuelas [11] pointed out that plants under drought tended to have high C:P ratio in leaves and litters.

Terrestrial plants change their C:N and N:P ratios in response to changes in N availability [20], [32]. N dynamics have been proved to drive stoichiometric shifts in both plant tissues and mineral soils [31]. It has, in fact, become a widely stated view that increasing soil N availability through N fertilization or atmospheric N deposition would increase N:P but decrease C:N ratios in plants [20], [25], [32], [39]. For instance, a meta-analysis demonstrated that N addition significantly increased the N:P but reduced the C:N ratios of the photosynthetic tissues of woody and herbaceous plants [20]. Our results were in accordance with these studies because N fertilization obtained higher increases in tissue N concentration than P concentration but had no visible effect on tissue C concentration (Figs. 1, 2 and 3). Although the responses of plant C:N and N:P ratios to N addition have been widely investigated, the response of C:P ratio to N addition has received less attention. We found that N application decreased tissue C:P ratio in the late growth period (Fig. 7), reflecting that N availability had significant impacts on P uptake (Fig. 3) but little effects on C assimilation (Fig. 1).

### Implication of the C:N:P stoichiometry for evaluating nutrient limitation in rice production systems

Plant C:N:P stoichiometry determines plant community composition and structure (resource allocation), trophic dynamics, and nutrient limitation [19], [20], [33]. Among those stoichiometric ratios, N:P ratio is the most widely investigated because it reflects important biochemical constraints on relative investments in N-rich proteins (approx. 16% by mass) and the P-rich ribosomal RNA used to generate them [19]. Though fluctuating across a broad range (approx. 1–100) in individual measurements [15], N:P ratio, with high diagnostic value for nutrient limitation [22], offers a simple and cost-effective tool for evaluating the limiting patterns of nutrient from individual plant species to terrestrial ecosystems [6], [19], [40], [41]. Based on theoretical considerations and laboratory results, the ‘Liebig’s law of the minimum’ for N and P suggests that there is a ‘critical (also called ideal or optimal) N:P ratio’ below which plant growth is limited only by N and above which growth is limited only by P, and within which growth is co-limited by both elements [15], [22], [40], [42]. Tremendous researches were carried out on this critical N:P ratio in natural vegetations. Koerselman & Meuleman [40] suggested an optimal aboveground biomass N:P ratio of 14–16 in wetland plant species by a review of 40 fertilization studies. Güsewell [15] proposed a broader range of 10–20 as the ideal N:P ratio in terrestrial plants from short-term fertilization experiments. Knecht & Göransson [42] sorted out an average optimal N:P ratio of about 10.0 in field-grown terrestrial plants from published data. Sadras et al. [22] reported that the critical N:P ratio was 4.5 for oilseed crops (n = 81), 5.6 for cereals (n = 134) and 8.7 for legumes (n = 52), and stated that over 40% of cereal and oilseed crops attaining maximum yield had N:P ratios in a relatively narrow range between 4 and 6. Aulakh & Malhi [43] summarized that the main cereal crops, such as rice, wheat, and corn, typically had optimal N:P ratios in both grain and straw in a fairly narrow range of 4.2–6.7. As for rice, Witt et al. [44] simulated balanced nutrient uptakes of 14.7 kg N and 2.6 kg P per ton of grain with a corresponding N:P ratio of 5.7. In this study, the rice aboveground plant N:P ratio (excluding N0 treatment) ranged from 4.4 to 6.4 through the growing seasons, which was somewhat lower than those critical N:P ratios in other plant species mentioned above (10–20) but still remained within the normal range (4.2–6.7), suggesting that the growth of rice in this region was co-limited by N and P. Collectively, these findings provided valuable insights into the mechanisms underpinning plant essential elements cycling in response to simultaneous changes in water and N availability, and broadened the knowledge of the C:N:P stoichiometry in subtropical high-yield rice systems.

## Supporting Information

File S1 **Table S1,** Combined analysis of variance (*F* values) for C, N and P concentrations in root, stem-leaf, and panicle of late-season rice at various growth stages under different water and N managements in 2010–2011. **Table S2,** Combined analysis of variance (*F* values) for C, N and P accumulation and partitioning in root, stem-leaf, and panicle of late-season rice at various growth stages under different water and N managements in 2010–2011. **Table S3,** Combined analysis of variance (*F* values) for C:N:P stoichiometric ratios in root, stem-leaf, and panicle of late-season rice at various growth stages under different water and N managements in 2010–2011.
(DOC)
