# Supplementary material for: Carbon, Nitrogen and Phosphorus Accumulation and Partitioning, and C:N:P Stoichiometry in Late-Season Rice under Different Water and Nitrogen Managements
Source: PLoS One. 2014 Jul 3;9(7):e101776. doi: 10.1371/journal.pone.0101776 (PMC4081737; doi:10.1371/journal.pone.0101776)
Supplement: File S1 — Table S1, Combined analysis of variance (F values) for C, N and P concentrations in root, stem-leaf, and panicle of late-season rice at various growth stages under different water and N managements in 2010–2011. Table S2, Combined analysis of variance (F values) for C, N and P accumulation and partitioning in root, stem-leaf, and panicle of late-season rice at various growth stages under different water and N managements in 2010–2011. Table S3, Combined analysis of variance (F values) for C:N:P stoichiometric ratios in root, stem-leaf, and panicle of late-season rice at various growth stages under different water and N managements in 2010–2011. (DOC) [file pone.0101776.s001.doc]

# Supporting Information

**Table S1** Combined analysis of variance (*F* values) for C, N and P concentrations in root, stem-leaf, and panicle of late-season rice at various growth stages under different water and N managements in 2010-2011 (DOC).

**Table S2** Combined analysis of variance (*F* values) for C, N and P accumulation and partitioning in root, stem-leaf, and panicle of late-season rice at various growth stages under different water and N managements in 2010-2011 (DOC).

**Table S3** Combined analysis of variance (*F* values) for C:N:P stoichiometric ratios in root, stem-leaf, and panicle of late-season rice at various growth stages under different water and N managements in 2010-2011 (DOC).

**Table S1** Combined analysis of variance (*F* values) for C, N and P concentrations in root, stem-leaf, and panicle of late-season rice at various growth stages under different water and N managements in 2010-2011.

| Element | Plant part | Source of variation | Growth stage | | | | |
| --- | --- | --- | --- | --- | --- | --- | --- |
|  |  |  | Seedling | Tillering | Booting | Filling | Maturity |
| C | Root | Water (W) | 0.43^ns^ | 9.17^**^ | 1.64^ns^ | 0.04^ns^ | 0.50^ns^ |
|  |  | Nitrogen (N) | 0.79^ns^ | 1.71^ns^ | 1.96^ns^ | 9.87^**^ | 28.90^**^ |
|  |  | W × N | 1.46^ns^ | 1.36^ns^ | 2.16^ns^ | 1.11^ns^ | 0.19^ns^ |
|  | Stem-leaf | Water (W) | 0.24^ns^ | 4.33^*^ | 5.32^*^ | 1.29^ns^ | 8.90^**^ |
|  |  | Nitrogen (N) | 0.53^ns^ | 3.02^*^ | 3.03^*^ | 2.29^ns^ | 1.17^ns^ |
|  |  | W × N | 0.34^ns^ | 0.05^ns^ | 0.32^ns^ | 0.45^ns^ | 0.43^ns^ |
|  | Panicle | Water (W) | - | - | - | 0.13^ns^ | 0.09^ns^ |
|  |  | Nitrogen (N) | - | - | - | 8.24^**^ | 1.29^ns^ |
|  |  | W × N | - | - | - | 1.10^ns^ | 0.67^ns^ |
|  |  |  |  |  |  |  |  |
| N | Root | Water (W) | 0.24^ns^ | 2.76^ns^ | 0.60^ns^ | 0.03^ns^ | 14.15^**^ |
|  |  | Nitrogen (N) | 0.07^ns^ | 78.11^**^ | 51.29^**^ | 142.58^**^ | 289.34^**^ |
|  |  | W × N | 0.59^ns^ | 2.34^ns^ | 0.30^ns^ | 0.21^ns^ | 1.68^ns^ |
|  | Stem-leaf | Water (W) | 0.04^ns^ | 0.32^ns^ | 0.78^ns^ | 0.03^ns^ | 1.54^ns^ |
|  |  | Nitrogen (N) | 0.67^ns^ | 117.21^**^ | 127.97^**^ | 96.84^**^ | 210.37^**^ |
|  |  | W × N | 0.32^ns^ | 0.87^ns^ | 0.51^ns^ | 1.27^ns^ | 0.36^ns^ |
|  | Panicle | Water (W) | - | - | - | 12.97^**^ | 2.01 |
|  |  | Nitrogen (N) | - | - | - | 54.75^**^ | 60.31^**^ |
|  |  | W × N | - | - | - | 2.02^ns^ | 0.38^ns^ |
|  |  |  |  |  |  |  |  |
| P | Root | Water (W) | 0.06^ns^ | 0.03^ns^ | 2.97^ns^ | 14.04^**^ | 40.24^**^ |
|  |  | Nitrogen (N) | 0.15^ns^ | 7.68^**^ | 3.65^*^ | 41.48^**^ | 58.84^**^ |
|  |  | W × N | 0.23^ns^ | 0.97^ns^ | 0.04^ns^ | 0.77^ns^ | 2.60^ns^ |
|  | Stem-leaf | Water (W) | 0.60^ns^ | 4.50^*^ | 14.93^**^ | 20.21^**^ | 56.13^**^ |
|  |  | Nitrogen (N) | 0.61^ns^ | 7.07^**^ | 2.92^*^ | 17.32^**^ | 17.14^**^ |
|  |  | W × N | 0.23^ns^ | 0.36^ns^ | 0.08^ns^ | 0.88^ns^ | 1.57^ns^ |
|  | Panicle | Water (W) | - | - | - | 9.79^**^ | 47.42^**^ |
|  |  | Nitrogen (N) | - | - | - | 13.63^**^ | 8.77^**^ |
|  |  | W × N | - | - | - | 1.81^ns^ | 0.59^ns^ |

^*^ Significant at *P* < 0.05.

^**^ Significant at *P* < 0.01.

^ns^ No significant.

**Table S2** Combined analysis of variance (*F* values) for C, N and P accumulation and partitioning in root, stem-leaf, and panicle of late-season rice at various growth stages under different water and N managements in 2010-2011.

| Element | Growth stage | Source of variation | Accumulation | | |  | Partitioning | |  |
| --- | --- | --- | --- | --- | --- | --- | --- | --- | --- |
|  |  |  | Root | Stem-leaf | Panicle |  | Root | Stem-leaf | Panicle |
| C | Seedling | Water (W) | 0.00^ns^ | 0.21^ns^ | - |  | 0.21^ns^ | 0.21^ns^ | - |
|  |  | Nitrogen (N) | 1.94^ns^ | 0.60^ns^ | - |  | 1.08^ns^ | 1.08^ns^ | - |
|  |  | W × N | 0.09^ns^ | 0.45^ns^ | - |  | 0.45^ns^ | 0.45^ns^ | - |
|  | Tillering | Water (W) | 1.33^ns^ | 2.86^ns^ | - |  | 0.14^ns^ | 0.14^ns^ | - |
|  |  | Nitrogen (N) | 8.97^**^ | 7.94^**^ | - |  | 1.01^ns^ | 1.01^ns^ | - |
|  |  | W × N | 0.59^ns^ | 0.13^ns^ | - |  | 0.26^ns^ | 0.26^ns^ | - |
|  | Booting | Water (W) | 4.75^*^ | 5.29^*^ | - |  | 8.96^**^ | 8.96^**^ | - |
|  |  | Nitrogen (N) | 32.25^**^ | 15.67^**^ | - |  | 0.99^ns^ | 0.99^ns^ | - |
|  |  | W × N | 2.34^ns^ | 0.09^ns^ | - |  | 0.82^ns^ | 0.82^ns^ | - |
|  | Filling | Water (W) | 10.09^**^ | 7.67^**^ | 0.85 ^ns^ |  | 17.67^**^ | 2.42^ns^ | 0.02^ns^ |
|  |  | Nitrogen (N) | 107.63^*^* | 84.22^**^ | 33.57^**^ |  | 7.28^**^ | 3.42^*^ | 1.53^ns^ |
|  |  | W × N | 0.88^ns^ | 0.43^ns^ | 0.17^ns^ |  | 0.75^ns^ | 0.10^ns^ | 0.10^ns^ |
|  | Maturity | Water (W) | 19.57^**^ | 3.94^ns^ | 58.37^**^ |  | 2.85^ns^ | 9.06^**^ | 7.95^**^ |
|  |  | Nitrogen (N) | 250.00^**^ | 473.35^**^ | 997.28^**^ |  | 15.07^**^ | 16.64^**^ | 38.29^**^ |
|  |  | W × N | 0.54^ns^ | 0.17^ns^ | 2.41^ns^ |  | 0.62^ns^ | 0.57^ns^ | 1.18^ns^ |
|  |  |  |  |  |  |  |  |  |  |
| N | Seedling | Water (W) | 0.01^ns^ | 0.41^ns^ | - |  | 0.45^ns^ | 0.45^ns^ | - |
|  |  | Nitrogen (N) | 2.01^ns^ | 1.13^ns^ | - |  | 0.82^ns^ | 0.82^ns^ | - |
|  |  | W × N | 0.19^ns^ | 0.12^ns^ | - |  | 0.36^ns^ | 0.36^ns^ | - |
|  | Tillering | Water (W) | 0.67^ns^ | 2.09^ns^ | - |  | 0.11^ns^ | 0.11^ns^ | - |
|  |  | Nitrogen (N) | 53.96^**^ | 84.10^**^ | - |  | 9.96^**^ | 9.96^**^ | - |
|  |  | W × N | 0.83^ns^ | 0.32^ns^ | - |  | 1.49^ns^ | 1.49^ns^ | - |
|  | Booting | Water (W) | 5.81^*^ | 6.38^*^ | - |  | 12.28^**^ | 12.28^**^ | - |
|  |  | Nitrogen (N) | 107.98^**^ | 104.87^**^ | - |  | 6.07^**^ | 6.07^**^ | - |
|  |  | W × N | 2.76^ns^ | 0.20^ns^ | - |  | 0.68^ns^ | 0.68^ns^ | - |
|  | Filling | Water (W) | 6.42^*^ | 4.88^*^ | 0.94 ^ns^ |  | 8.36^**^ | 6.95^*^ | 2.82^ns^ |
|  |  | Nitrogen (N) | 231.33^**^ | 177.45^**^ | 89.28^**^ |  | 2.13^ns^ | 340.21^**^ | 1.42^ns^ |
|  |  | W × N | 0.72^ns^ | 0.72^ns^ | 0.19^ns^ |  | 0.07^ns^ | 0.20^ns^ | 0.20^ns^ |
|  | Maturity | Water (W) | 0.85^ns^ | 1.81^ns^ | 35.39^**^ |  | 3.58^ns^ | 7.04^*^ | 12.04^**^ |
|  |  | Nitrogen (N) | 522.11^**^ | 1002.97^**^ | 514.70^**^ |  | 29.84^**^ | 91.95^**^ | 145.14^**^ |
|  |  | W × N | 0.87^ns^ | 0.28^ns^ | 1.73^ns^ |  | 1.38^ns^ | 0.74^ns^ | 1.47^ns^ |
|  |  |  |  |  |  |  |  |  |  |
| P | Seedling | Water (W) | 0.09^ns^ | 0.08^ns^ | - |  | 0.62^ns^ | 0.62^ns^ | - |
|  |  | Nitrogen (N) | 1.60^ns^ | 0.50^ns^ | - |  | 1.29^ns^ | 1.29^ns^ | - |
|  |  | W × N | 0.09^ns^ | 0.50^ns^ | - |  | 0.62^ns^ | 0.62^ns^ | - |
|  | Tillering | Water (W) | 2.71^ns^ | 6.73^*^ | - |  | 0.04^ns^ | 0.04^ns^ | - |
|  |  | Nitrogen (N) | 9.33^**^ | 13.67^**^ | - |  | 0.41^ns^ | 0.41^ns^ | - |
|  |  | W × N | 1.67^ns^ | 0.48^ns^ | - |  | 0.63^ns^ | 0.63^ns^ | - |
|  | Booting | Water (W) | 0.28^ns^ | 17.85^**^ | - |  | 4.73^*^ | 4.73^*^ | - |
|  |  | Nitrogen (N) | 19.77^**^ | 18.52^**^ | - |  | 1.33^ns^ | 1.33^ns^ | - |
|  |  | W × N | 0.92^ns^ | 0.08^ns^ | - |  | 0.67^ns^ | 0.67^ns^ | - |
|  | Filling | Water (W) | 2.46^ns^ | 28.04^**^ | 5.78^*^ |  | 2.33^ns^ | 1.27^ns^ | 0.11^ns^ |
|  |  | Nitrogen (N) | 65.67^**^ | 110.01^**^ | 37.36^**^ |  | 7.52^**^ | 4.54^*^ | 1.01^ns^ |
|  |  | W × N | 0.33^ns^ | 0.38^ns^ | 0.45^ns^ |  | 0.31^ns^ | 0.14^ns^ | 0.26^ns^ |
|  | Maturity | Water (W) | 4.57^*^ | 35.13^**^ | 3.01^*^ |  | 0.43^ns^ | 10.80^**^ | 9.60^**^ |
|  |  | Nitrogen (N) | 163.19^**^ | 273.58^**^ | 234.30^**^ |  | 25.72^**^ | 8.74^**^ | 12.44^**^ |
|  |  | W × N | 2.27^ns^ | 1.60^ns^ | 0.10^ns^ |  | 1.14^ns^ | 0.41^ns^ | 0.81^ns^ |

^*^ Significant at *P* < 0.05.

^**^ Significant at *P* < 0.01.

^ns^ No significant.

**Table S3** Combined analysis of variance (*F* values) for C:N:P stoichiometric ratios in root, stem-leaf, and panicle of late-season rice at various growth stages under different water and N managements in 2010-2011.

| Ratio | Plant part | Source of variation | Growth stage | | | | |
| --- | --- | --- | --- | --- | --- | --- | --- |
|  |  |  | Seedling | Tillering | Booting | Filling | Maturity |
| C:N | Root | Water (W) | 0.61^ns^ | 0.03^ns^ | 0.07^ns^ | 0.04^ns^ | 6.33^*^ |
|  |  | Nitrogen (N) | 0.24^ns^ | 85.71^**^ | 54.03^**^ | 152.02^**^ | 353.31^**^ |
|  |  | W × N | 0.97^ns^ | 1.69^ns^ | 0.56^ns^ | 0.72^ns^ | 0.45^ns^ |
|  | Stem-leaf | Water (W) | 0.06^ns^ | 0.14^ns^ | 0.00^ns^ | 0.12^ns^ | 4.01^*^ |
|  |  | Nitrogen (N) | 0.74^ns^ | 171.80^**^ | 169.96^**^ | 129.32^**^ | 294.14^**^ |
|  |  | W × N | 0.35^ns^ | 1.16^ns^ | 0.46^ns^ | 0.60^ns^ | 0.25^ns^ |
|  | Panicle | Water (W) | - | - | - | 1.78^ns^ | 14.83^**^ |
|  |  | Nitrogen (N) | - | - | - | 79.59^**^ | 50.97^**^ |
|  |  | W × N | - | - | - | 1.57^ns^ | 0.18^ns^ |
|  |  |  |  |  |  |  |  |
| N:P | Root | Water (W) | 0.02^ns^ | 1.78^ns^ | 4.09^*^ | 14.62^**^ | 8.71^**^ |
|  |  | Nitrogen (N) | 0.26^ns^ | 33.90^**^ | 17.44^**^ | 28.45^**^ | 104.79^**^ |
|  |  | W × N | 0.39^ns^ | 3.08^*^ | 0.27^ns^ | 0.36^ns^ | 1.24^ns^ |
|  | Stem-leaf | Water (W) | 0.23^ns^ | 2.43^ns^ | 7.89^**^ | 14.40^**^ | 99.15^**^ |
|  |  | Nitrogen (N) | 1.79^ns^ | 284.04^**^ | 101.64^**^ | 65.76^**^ | 205.48^**^ |
|  |  | W × N | 0.63^ns^ | 3.02^*^ | 0.59^ns^ | 0.94^ns^ | 2.33^ns^ |
|  | Panicle | Water (W) | - | - | - | 55.96^**^ | 34.32^**^ |
|  |  | Nitrogen (N) | - | - | - | 41.24^**^ | 12.24^**^ |
|  |  | W × N | - | - | - | 1.85^ns^ | 0.53^ns^ |
|  |  |  |  |  |  |  |  |
| C:P | Root | Water (W) | 0.18^ns^ | 2.11^ns^ | 4.83^*^ | 15.82^**^ | 44.87^**^ |
|  |  | Nitrogen (N) | 0.32^ns^ | 4.60^*^ | 2.74^ns^ | 37.64^**^ | 33.11^**^ |
|  |  | W × N | 0.44^ns^ | 1.62^ns^ | 0.33^ns^ | 0.75^ns^ | 2.02^ns^ |
|  | Stem-leaf | Water (W) | 0.68^ns^ | 2.91^ns^ | 9.83^**^ | 19.13^**^ | 53.49^**^ |
|  |  | Nitrogen (N) | 0.58^ns^ | 6.93^**^ | 2.81^ns^ | 16.77^**^ | 19.96^**^ |
|  |  | W × N | 0.31^ns^ | 0.54^ns^ | 0.09^ns^ | 0.86^ns^ | 1.34^ns^ |
|  | Panicle | Water (W) | - | - | - | 9.83^**^ | 40.81^**^ |
|  |  | Nitrogen (N) | - | - | - | 12.62^**^ | 6.27^**^ |
|  |  | W × N | - | - | - | 1.44^ns^ | 0.26^ns^ |

^*^ Significant at *P* < 0.05.

^**^ Significant at *P* < 0.01.

^ns^ No significant.
